# Solid Particle Number Emission Factors of Euro VI Heavy-Duty Vehicles on the Road and in the Laboratory

**DOI:** 10.3390/ijerph15020304

**Published:** 2018-02-09

**Authors:** Barouch Giechaskiel

**Affiliations:** Joint Research Centre–European Commission, via E. Fermi 2749, 21027 Ispra, Italy; barouch.giechaskiel@ec.europa.eu; Tel.: +39-0332-78-5312

**Keywords:** particle measurement program (PMP), real driving emissions (RDE), trucks, diesel particulate filter (DPF), regeneration, portable emission measurement systems (PEMS), compressed natural gas (CNG), ultrafine particles

## Abstract

Particulate matter (PM), and in particular ultrafine particles, have a negative impact on human health. The contribution of vehicle PM emissions to air pollution is typically quantified with emission inventories, which need vehicle emission factors as input. Heavy-duty vehicles, although they represent a small percentage of the vehicle population in nearly every major country, contribute the majority of the on-road PM emissions. However, the published data of modern heavy-duty vehicle emissions are scarce, and for the newest Euro VI technologies, almost non-existent. The main objective of this paper is to present Solid Particle Number (SPN) emission factors from Euro VI heavy-duty vehicles using diesel, Compressed Natural Gas (CNG), or Liquefied Natural Gas (LNG). Urban, rural and motorway (highway) emissions were determined on the road at various European cities using SPN Portable Emission Measurement Systems (PEMS). Additional tests on a heavy-duty chassis dynamometer showed that the solid sub-23 nm fraction, which is not covered at the moment in the European regulation, is high, especially for CNG engines. The significant contribution of regeneration events and the effect of ambient temperature and engine cold-start on particle emissions were also discussed.

## 1. Introduction

### 1.1. PM, Health Effects, and Emission Models

Particulate matter (PM) consists of a complex mixture of solid and liquid particles of organic and inorganic substances suspended in the air. PM causes damage to ecosystems and cultural sites, and reduced visibility. Air pollution, and in particular PM_2.5_ (smaller than 2.5 μm) is now clearly recognized as an important global risk factor for disease [1].

Ultrafine particles (smaller than 0.1 μm) have been associated with short-term cardiorespiratory and central nervous system adverse health effects [2]. Clinical and toxicological studies have shown that ultrafine particles can act through mechanisms not shared with larger particles [2]. Ultrafine particle have higher deposition fraction, deeper penetration, and higher retention rate in the lungs [3]. The higher surface area to mass ratio enables them to carry other surface adhered hazardous substances that can generate reactive oxygen species, the so-called “Trojan horse” effect [4]. Ultrafine particles can translocate from the lungs to other organs, for instance the heart and the brain [5,6]. The surface characteristics, the chemical composition, the biological components, and the solubility determine the response and the health outcomes [7,8]. Ultrafine particles are typically measured with particle number (PN) [9] or surface area instruments [10,11].

Anthropogenic PM sources include agriculture, waste incineration, energy production, domestic heating, road transport, and, brakes, tires and road wear. Road traffic contributes around 11–25% to the PM concentrations in Europe [12,13], higher in Asia [13,14], and can reach >50% in some cities [15]. Particularly heavy-duty vehicles, which represent <5% of the vehicle population in nearly every major country, contribute 40–60% of the road-traffic PM emissions [16]. A study estimated that trucks and buses within New York City accounted for the largest share of on-road mobile-attributable ambient PM_2.5_ and contributed 53% of the PM_2.5_-attributable deaths [17]. The contribution of road traffic in terms of other metrics such as black carbon is larger [18]. For PN the traffic contribution can be even higher (60%) due to the high number of nuclei particles formed from the unburnt fuel and lubricant or secondary formation, reaching 90% in busy roads [19] or even 99% in tunnels [20]. 

The greenhouse gas emission reduction policies and the goal to keep the global temperature increase below 2 °C commits the European Union (EU) to reduce emissions by at least 20% below 1990 levels by 2020, and by 80–95% by 2050 [21]. The Transport White Paper [22] sets out how the transport system can reduce its emissions by 60% in the same period. Regarding urban transport, the target is 50% shift away from conventionally fueled cars by 2030, phasing them out in cities by 2050. Thus, a big shift to cleaner cars and cleaner fuels is required. Similar policies are followed all over the world [23,24,25].

The contribution of vehicle emissions to air pollution is typically quantified with emission inventories [26]. Emissions are estimated using various calculation tools (models) (e.g., COPERT (Computer Programme to calculate emissions from road transport) [27], HBEFA (Handbook Emission Factors for Road Transport) [28], MOVES (Motor Vehicle Emission Simulator) [29]). Generally, vehicle fleet and activity data with the respective emission factors are combined. These models are increasingly being used not only for emission inventorying purposes but also to assess air quality policies, produce emission projections, and to set environmental targets (e.g., [30]). These tools use vehicle emission factors as default values, but they are frequently updated using experimental results [31]. Different experimental methods can be used, such as remote sensing, chassis dynamometer measurements, and on-road testing with portable systems (for details see [32] and references therein). Remote sensing for PN has not been applied yet and experimental data for PN emissions are limited, especially for the newest Euro VI heavy-duty vehicles [33,34]. 

### 1.2. PN Regulation in EU

Based on the findings of the Particle Measurement Program (PMP) [35], the European emissions regulation requires, in addition to PM mass, the measurement of Solid Particle Number (SPN) > 23 nm (for details see Section 2) for type approval of diesel light-duty vehicles since 2011 (Euro 5b) [36], and for Gasoline Direct Injection (GDI) light-duty vehicles since 2014 (Euro 6) [37]. Real Driving Emissions (RDE) testing on the road with Portable Emissions Measurement Systems (PEMS) for SPN and NO_x_ during type approval and in-service conformity testing was recently (in 2017) introduced for light-duty vehicles [38].

Regarding heavy-duty vehicles the type approval of an engine is conducted in a test bed following a prescribed test cycle where the engine rpm and torque are varied. The test is conducted twice: With engine starting with coolant and oil temperature at ambient conditions (20–30 °C) and the engine starting warmed up (the results are weighted 14% and 86% respectively). This engine then can be used for various applications for instance in a truck (category N) or a bus (category M). The SPN limit for heavy-duty engines (6 × 10^11^ p/kWh) (“p” will be used for particles from now on) was introduced in 2013 (Euro VI) for compression ignition (diesel) engines [39] and in 2014 for positive ignition engines [40]. The SPN measurement procedure is almost identical to the light-duty vehicles procedure [41,42].

The in-service conformity (ISC) (or in-use compliance in the USA) testing of a heavy-duty engine was conducted in the test bed by removing the engine from a vehicle in normal use. Since Euro VI vehicles, the testing is conducted on the road over normal driving patterns, conditions and payloads using PEMS [39]. The testing is conducted over a mix of urban (≤50 km/h), rural, and motorway (highway) (>75 km/h) conditions, with exact percentages of these conditions depending on vehicle category [43]. The first in-use test should be conducted at the time of type approval testing and the result should be lower than the Euro VI limit corrected with a conformity factor (1.5 for gaseous pollutants) that takes into account the PEMS measurement uncertainty. Although the PEMS testing is applicable for both gaseous and PM emissions in US, in Europe it is currently applicable to gaseous emissions only. After a long evaluation of the PM mass method with PEMS [44], in 2015 it was decided to evaluate the SPN method, which should be introduced in 2019.

### 1.3. Open Points for SPN Emissions

As heavy-duty engines are type approved in a test bed and SPN PEMS were only recently introduced, the real world SPN emissions of modern vehicles are not well known [45]. The limited information available today is based on studies in the USA with a mobile dilution tunnel (e.g., [46]) or systems that mimic the regulated SPN procedure (e.g., [47]). As ISC testing should cover an ambient temperature range from −7 °C to +35 °C, there is a need to better understand the influence of low (or even high) ambient temperatures on SPN. The knowledge of the ambient temperature effect on SPN emissions is based mainly on light-duty vehicle studies [45,48,49], because the facilities worldwide (heavy-duty engine test beds or chassis dynamometers) that can modify the room (ambient) temperature are limited. In general, emissions are higher at lower temperatures, but when the engine is hot the ambient temperature plays a minor role, as long as the combustion strategy remains the same [49,50]. Another topic under investigation is the introduction of (engine) cold start for the ISC testing (i.e., emissions until the coolant temperature reaches 70 °C). Many studies have shown that gaseous emissions are higher during the first minutes of testing due to inefficient operation of the after-treatment devices and the higher engine out emissions (e.g., [51,52]). However, for SPN emissions the trend is not clear. Usually, higher SPN emissions are measured [35], but not always [52].

Another topic under discussion is the existence of solid sub-23 nm particles that the current regulated methodology does not measure [53]. Recent studies have shown that during urea injection not only the >23 nm SPN concentration increases [54], but also the ratio of 10 nm to 23 nm SPN concentrations is higher [55]. For CNG engines most studies have shown that the majority of particle emissions is <23 nm [56], raising concerns for the regulated method.

Regeneration of the DPF to remove the collected soot results in elevated emissions during passive or active regeneration [47,57]. However, the contribution of these emissions to the emissions between regenerating events is not clear (i.e., the regeneration adjustment factor [42]).

### 1.4. Objectives of the Paper

The main objective of this paper is to present SPN emission factors of modern (i.e., Euro VI type approved) heavy-duty vehicles based on on-road measurements with SPN PEMS. Additionally, input on topics that haven’t been covered adequately in the literature will be given; namely correlation of laboratory and on-road SPN emissions, effect of cold start and ambient temperature on SPN emissions, existence of sub-23 nm solid particles, and contribution of regeneration emissions on the SPN emissions.

## 2. Materials and Methods

The vehicles were tested in one laboratory and/or on the road at various European cities. Some data were extracted from published data.

### 2.1. Vehicles and Fuels

The test fleet consisted of trucks (N category) or buses (M category) using EN590 diesel or natural gas. Natural gas can be stored either compressed at pressures over 200 bar (Compressed Natural Gas, CNG) or cryogenically in liquid form at temperatures approximately −162 °C (Liquefied Natural Gas, LNG). All vehicles complied to the Euro VI standards (i.e., the cold and hot start weighted SPN emissions of the engine were <6 × 10^11^ p/kWh). Details of the vehicles can be found in Table 1. The diesel vehicles were all equipped with Diesel Oxidation Catalyst (DOC), DPF, Selective Catalytic Reduction (SCR) for NO_x_ systems and ammonia slip catalyst. The CNG vehicles were stoichiometric with three-way catalyst (TWC). More information can be found in the cited literature.

The experimental details are summarized in Table 2 and will be described in the next sections. The LNG data were taken from The Netherlands Organization for Applied Scientific Research (TNO) [58], some of the diesel vehicles’ data were taken from the Swedish Transport Agency (STA) [59], or published papers [45,52]. Some data were provided by the European Automobile Manufacturers' Association (ACEA) for the SPN PEMS project [60]. The rest of the vehicles were tested during various projects such as the evaluation of SPN PEMS [60], cold start emissions, or tools for calculating CO_2_ emissions [61] at European Commission’s Joint Research Centre (JRC, Ispra, Italy). None of the vehicles was optimized for low SPN emissions and in some cases on purpose “worst cases” were tested to challenge the instrumentation. Thus, the results of this paper should be representative of the current Euro VI heavy-duty vehicles. The tests were conducted in the 2016–2017 period (except the STA tests in 2014 and DPF N3 #3 in 2015).

For all tests commercially available fuels were used (diesel fuel with <10 ppm sulfur content). The LNG consisted of 92% methane [58], while the commercially available CNG typically consisted of 77% methane (CNG N1 #3) [59], or >87% for the rest CNG vehicles [42].

### 2.2. Laboratory Testing

The laboratory tests were conducted on the 2-axis roller dynamometer of the Vehicle Emissions Laboratory (VELA 7) of JRC. The dyno settings were not available from the vehicle manufacturers and for this reason realistic estimated values were used [61]. The test mass was set to simulate approximately 50–60% payload. For the tests described here, the climatic room temperature varied between −7 °C and +35 °C, but typically temperatures between 20 °C and 25 °C were used. The exhaust gas was connected to the full dilution tunnel with a 9 m tube (the last 4 m insulated). The full dilution tunnel with constant volume sampling (CVS) was used with flow rates of 100–120 m^3^/min depending on the engine size. With this flow rate at least a dilution ratio of 5:1 was achieved, even for the bigger engines.

A SPN measurement system (AVL Particle Counter (APC) 489, Graz, Austria), compliant with the heavy-duty engines [42] regulations, was used at the dilution tunnel (CVS) [62]. The Volatile Particle Remover (VPR) of the system consisted of a hot dilution of approximately 25:1 at 150 °C, an evaporation tube at 350 °C and a final dilution in a porous diluter (10:1) with room temperature filtered air. The system was calibrated by the manufacturer and the dilution, which includes the particle losses (as average of 30 nm, 50 nm and 100 nm), is called Particle number Concentration Reduction Factor (PCRF) (approximately 250 in this study). Downstream of the VPR a butanol Condensation Particle Counter (CPC) (model TSI 3790) with 50% counting efficiency at 23 nm (d_50%_ = 23 nm) was measuring solid particles. In parallel, a butanol CPC (model TSI 3772) (d_50%_ =10 nm) was used in some tests to investigate the presence of particles between 10 and 23 nm. The additional losses below 30 nm were taken into account for the 10 nm CPC results as described in [53], i.e., a correction factor of 1.7 was applied. An Engine Exhaust Particle Sizer (EEPS) (model 3090 from TSI, Shoreview, MN, USA) was used directly at the dilution tunnel (CVS) for some tests with DPF N3 #4b to measure total PN (volatiles and solids). The repeatability of the SPN method (not the variability of the vehicles) is expected to be better than 15% [35,63].

**Figure 1:**
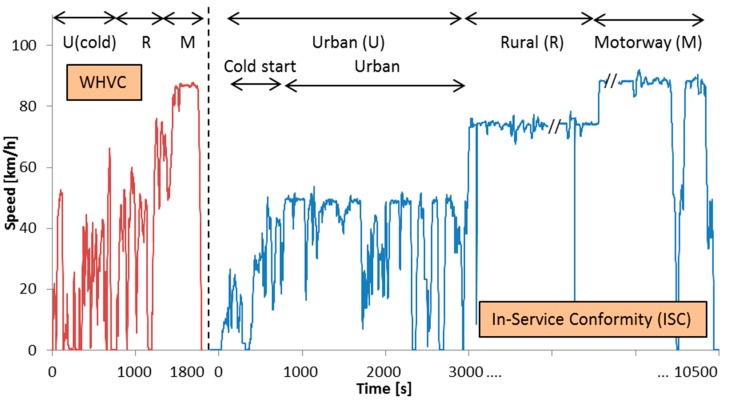
Test cycles: WHVC (World Harmonized Vehicle Cycle) and an example of an ISC (In-Service Conformity) cycle (plotted in the same figure for illustrative purposes). Note that parts of the rural and motorway phases have been cut for better visualization.

Some vehicles were tested under cold and hot engine start conditions with the Worldwide Harmonized Vehicle Cycle (WHVC), which was developed based on the same set of data used for the development of the engine type approval Worldwide Harmonized Test Cycle (WHTC) [64]. Unlike the WHTC, however, the WHVC is not used for regulatory testing. No slopes were added in most of the cases, thus the work of this cycle in many cases was different to the type approval work of the WHTC. The duration of the WHVC test is 1800 s. The first 900 s represent urban driving, the next 481 s rural, and the last 419 s motorway (highway). A graphical presentation of the cycle can be found in Figure 1. When the test was starting with cold engine (i.e., engine and coolant at ambient temperature), the urban phase was considered as “cold (urban) start”.

Some vehicles were tested in the laboratory with pre-determined ISC (In-Service-Conformity) cycles (approximately 150 km long with >2.5 h duration). For N3 vehicles the cycle consisted of urban (20%), rural (25%) and motorway (55%) phases in this order (excluding idling time) [43]. For the N2 vehicles the shares were urban (45%), rural (25%) and motorway (30%). The rural and motorway phases included short sections with low speeds (≤50 km/h); to simulate for example when stopping at tolls or crossing a village (see e.g., Figure 1). When the test was starting with cold engine (i.e., at ambient temperature), the first 900 s were separately evaluated to estimate the cold start (urban) emissions. In this case, the rest urban part was used for the evaluation of the (hot) urban emissions. The reason of selecting a duration of 900 s instead of a coolant temperature threshold was that the coolant temperature was not available for all vehicles in this paper. For vehicles that the coolant temperature was available, it was confirmed that at 900 s the coolant temperature was >75 °C. Additionally for vehicles that both the WHVC and ISC were available, it was noticed that the coolant temperature increase was similar at the two cycles. 

### 2.3. On-Road Testing

For the on-road tests portable SPN systems were used. The SPN PEMS were the modified TSI NPET (Nanoparticle Emission Tester) from HORIBA (Kyoto, Japan) or the commercial version OBS-ONE from HORIBA (almost identical systems) [65]. The first diluter (10:1) was located directly at the sample probe at the tailpipe. With a 4 m heated line at 60 °C the diluted aerosol was brought to the main cabinet where a heated catalytic stripper at 350 °C removed the volatile and semi-volatile particles. A second dilution (10:1) cooled down the aerosol and brought the concentration to the measuring range of the isopropyl alcohol-based CPC with d_50%_ at 23 nm (a TSI CPC 3007 with modified saturator and condenser temperatures). The SPN-PEMS and the PMP system were compared to each other periodically and the differences were found within 35% [60].

Depending on the vehicle, 4 or 5 inches exhaust flowmeters were used. For some vehicles the exhaust flowmeters were compared to the estimated exhaust flow rate from the difference between the total flow of the dilution tunnel and the dilution air flow and the differences were within 4%.

The on-road ISC cycles were similar to the ISC lab tests but not identical. A graphical presentation of a sample cycle can be found in Figure 1.

### 2.4. Literature and Received Data

The ACEA, TNO [58], and literature [45,52] received data were on-road tests that followed the procedures described above. The STA [59] data were extracted from a report. Cold and hot WHVCs were conducted at AVL MTC (Motortestcenter) AB (Stockholm, Sweden) using a similar setup as described previously for JRC.

### 2.5. Calculations

The trip was divided in “cold (urban) start” (first 900 s) (if the engine was cold), “urban” with hot engine (speeds ≤50 km/h), “rural” (speeds 50–75 km/h), and “motorway” phases (speeds >75 km/h). The rural and motorway phases included time periods with speeds <50 km/h and <75 km/h respectively (e.g., crossing a village, stopping at tolls). For the calculations these short section with low speeds were included in the rural and motorway phases respectively (i.e., they were not excluded or transferred to urban emissions). See Figure 1 as an example.

After time alignment of the signals, the SPN concentration of the system at the dilution tunnel was multiplied with the CVS flow rate, while the SPN PEMS at the tailpipe (both in the laboratory and on the road) was multiplied with the exhaust flow rate. The sum of the instantaneous particle number emissions during the urban, rural and motorway phases were divided with the respective distances to obtain the distance specific SPN emissions [p/km]. All emission events were included in the calculations (integrated emissions) and no parts were excluded as required in the regulations (e.g., <10% max power in Europe [39,43] and the Not-To-Exceed (NTE) concept in USA). When total trip emissions are reported, they were calculated weighing 14% the cold start emissions and 86% the mean value of hot start urban, rural, and motorway operation. Note that this approach is not the regulated one and gives higher weight to the urban emissions. Note also that the regulation requires emissions in p/kWh; in this paper results are expressed in p/km because they are more useful for emission factor models.

## 3. Results

**Figure 2:**
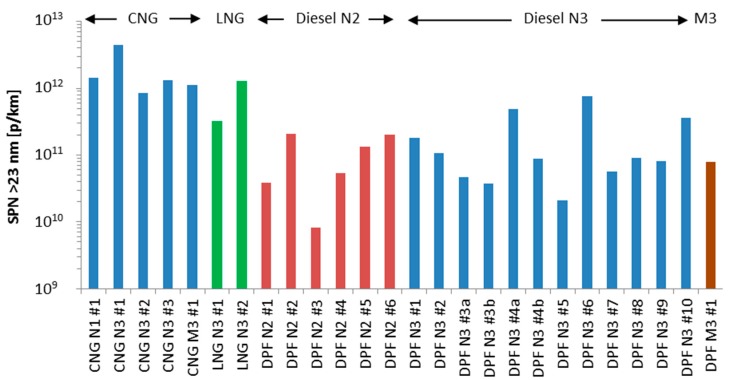
Weighted SPN emissions of test fleet (14% cold start, 86% mean of urban, rural, motorway). The small letters “a” and “b” indicate testing of the same vehicle after approximately 3–6 months.

The weighted emissions of the vehicles (14% cold start emissions and 86% mean value of urban, rural, and motorway emissions) are presented in Figure 2. The CNG and LNG vehicles range from 3.3 × 10^11^ to 4.5 × 10^12^ p/km. One of the LNG vehicles is lower than the assumed SPN limit 6 × 10^11^ p/km. The rest vehicles are higher by a factor of 1.4 to 2.4, with one exception (CNG N3 #1: 4.5 × 10^12^ p/km), that is 7.5 times higher. The specific vehicle had <3000 km and the lubricant contribution could be higher. The diesel vehicles (all with DPFs) ranged from 8 × 10^9^ to 7 × 10^11^ p/km. No particular trend can be seen for the different categories (trucks or buses, N2 or N3). 

**Figure 3:**
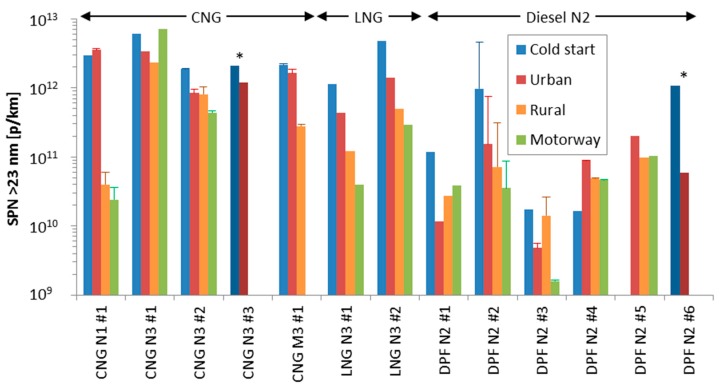

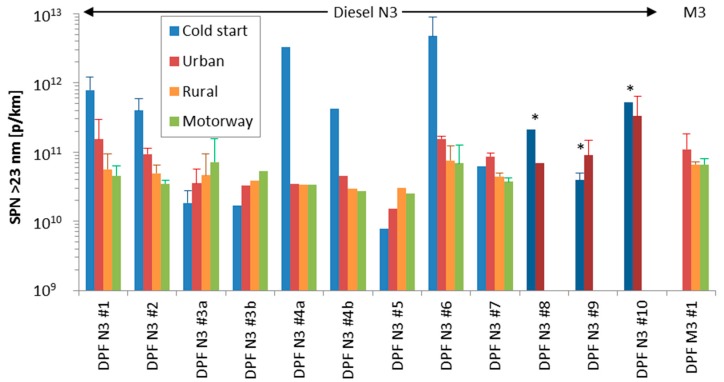
Urban cold start, urban hot, rural and motorway SPN emissions. Upper panel for CNG, LNG and diesel N2 vehicles. Lower panel for diesel N3 and M3 vehicles. Asterisk * indicates that the two columns refer to the complete WHVC (cold or hot). Error bars show the maximum value when 2 or more repetitions were available. The small letters “a” and “b” indicate testing of the same vehicle after approximately 3–6 months.

Figure 3 compares the urban SPN emissions with the engine cold or hot (i.e., after 900 s, coolant temperature >75 °C), rural, and motorway (hot) SPN emissions. In general, the emissions are higher when the engine is at ambient temperature (cold), however, not always, especially when the emission levels are <10^11^ p/km (see for example DPF N2 #4, DPF N3 #3, #7, #9). The rural and motorway emissions are in general lower than the urban emissions with a few exceptions (e.g., CNG N3 #1, DPF N2 #1, DPF N3 #3a). 

The results are based on 1–4 repetitions per vehicle (Figure 3). When 2 or more repetitions were available, the variability (expressed as ratio of maximum value to average minus one) was on average 35% (5–85%) with one exception which exceeded 300% (DPF N2 #2). Comparison of the mean emissions of the vehicles that were tested twice (DPF N3 #3 and #4, repetitions “a” and “b” in Figure 3) gave differences within 35% (except cold start >600%).

## 4. Discussion

The main objective of this paper was to present SPN emission factors of Euro VI heavy-duty vehicles. Results from 24 diesel, CNG, and LNG vehicles were presented (8 of them from the literature). 

### 4.1. Emission Levels

**Figure 4:**
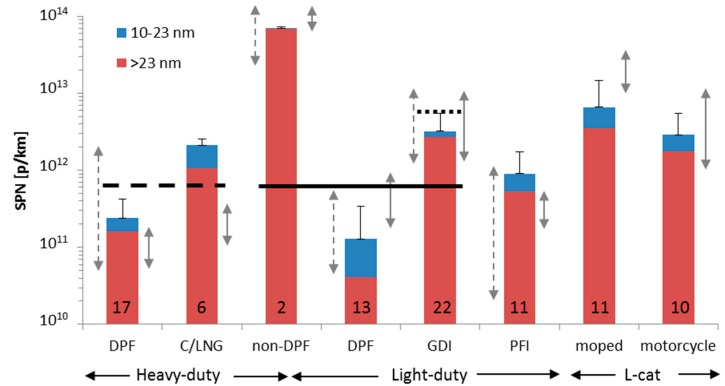
Overview of emission levels of different current vehicle categories, based on this study for the heavy-duty vehicles and the literature for the rest [53,66,67]. Dashed arrows on the left of the bars show reported range of an older SPN review [35]. Arrows on right shows suggested emission factors based on [68]. Error bars show one standard deviation (only positive side) for the number of vehicles shown in each bar. Horizontal lines give the European regulated SPN limits for SPN >23 nm. Note that for the GDIs of this figure the limit was 6 × 10^12^ p/km (dotted line). The dashed line shows a limit of 6 × 10^11^ p/km. However, the SPN limit applies only to heavy-duty engines (not vehicles) and is expressed in p/kWh. All tests at temperatures around 23 °C.

The emission levels of Figure 2 are summarized in Figure 4 for diesel and natural gas vehicles (cold start weighted 14%). To put the emission levels into perspective Figure 4 summarizes also emission levels of various vehicle categories based on a few studies that focused on the most recent technologies: Euro 5 and Euro 6 light-duty vehicles [53,66] (cold start included in the test cycle), Euro 2 and Euro 3 mopeds and motorcycles [67] (cold start weighted 30%). Emission factors from COPERT and HBEFA [33,34,68] and ranges from the latest SPN emissions review are also plotted [35]. The diesel vehicles have the highest emissions (>3 × 10^13^ p/km), while those equipped with DPF the lowest (<6 × 10^11^ p/km), in agreement with the literature (e.g., [69,70,71]). The spark ignition vehicles are on the order of 10^12^ p/km; lower for gasoline Port-Fuel Injection (PFI) vehicles, but higher for Gasoline Direct Injection (GDI) vehicles, mopes and motorcycles.

The emissions of the DPF equipped heavy-duty vehicles are similar with the range reported in a review paper [35] for older DPF equipped vehicles (5 × 10^10^–2 × 10^12^ p/km), retrofitted vehicles [46,71] or the COPERT and HBEFA estimated emission factors (5 × 10^10^–1.6 × 10^11^ p/km) [33,34]. This indicates that newer DPFs with or without catalytic washcoat alone or in combination with NO_x_ reduction systems remain very efficient. The emissions are much lower than heavy-duty diesel vehicles without DPF, which are in the range of 2 × 10^13^–2 × 10^14^ p/km [35,69]. The SPN emissions of the natural gas engines (3.3 × 10^11^ to 1.5 × 10^12^ p/km excluding CNG N3 #1) were higher than the DPF equipped vehicles and similar to what has been reported for Euro V CNG buses [72]. Thus, although natural gas vehicles emit virtually no visible PM or black soot and have less PM mass emissions than non-DPF equipped diesel vehicles [69], this is not necessarily true if they are compared to DPF equipped diesel vehicles for PM [73] and SPN (this study). As expected, the emission levels of LNG and CNG vehicles were similar because the combustion of the two forms of natural gas is identical: LNG is first vaporized and then injected, in a similar manner to CNG. The difference between the two is only the way that the fuels are stored on board the vehicle.

To give indications of the concentration levels emitted from the tailpipe, for example, a vehicle with cold start emissions of 3 × 10^12^ p/km, would have SPN concentration (for a few minutes) around 5 × 10^6^ p/cm^3^. For emission levels of 5 × 10^10^ p/km the concentration is between 1 × 10^3^ p/cm^3^ and 1 × 10^4^ p/cm^3^. A vehicle that would emit close to the ambient background level (measured at JRC laboratory ambient air [60] 4000 p/cm^3^ solid particles >23 nm), would have emissions levels between 2 × 10^10^ p/km and 1 × 10^11^ p/km depending on the engine size and the phase of the route (urban, rural, motorway). 

These results should be interpreted with care because they are not based on an extensive research of the literature and haven’t been weighted for the market shares of the evaluated vehicles. In addition, and in particular for the results of this paper, many parameters influence the repeatability (variability) of the reported SPN emission factors, such as instrumentation, DPF fill state, regeneration, test cycle and ambient temperature. For example, the uncertainty of the PMP system in the lab is on the order of 15%, while for the PEMS on the road at least 35% [60]. The rest influencing parameters (for the heavy-duty vehicle results presented in this paper) will be discussed in the next sections.

### 4.2. Engine Cold Start 

The enrichment of the air/fuel mixture during cold-start engine operation or at low ambient temperatures, in order to compensate for the reduced fuel vaporization and elevated engine components friction, leads to incomplete fuel combustion [74]. The higher engine out emissions combined with the lower efficiency of the after-treatment devices, as they haven’t reached their normal operating temperature range, result in higher emissions (Figure 3: compare urban cold start and urban hot). The total particles emitted during cold start (first 900 s) of the vehicles of this study were on average 3.4 × 10^12^ particles.

**Figure 5:**
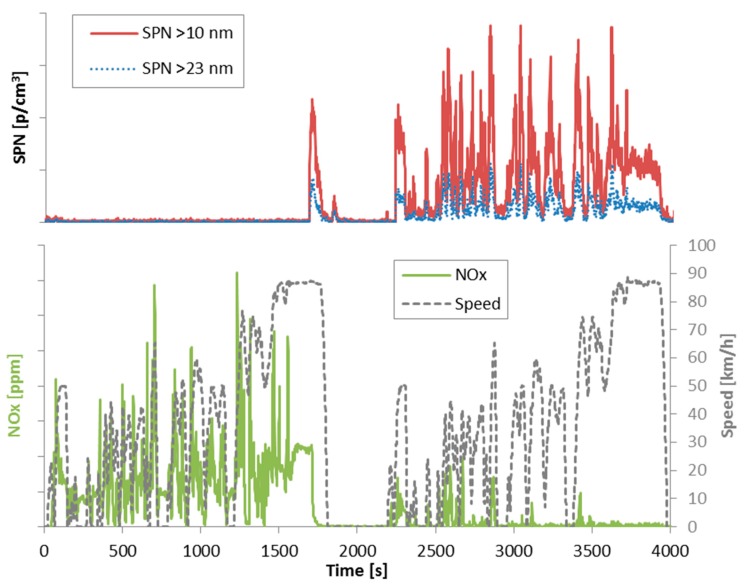
**Lower panel**: NO_x_ emissions during two WHVCs (first one with cold engine start). **Upper panel**: SPN emissions of the two WHVCs. Vehicle DPF N2 #3.

However, the SPN cold start emissions are not always higher than the hot emissions, especially when the emission levels are <10^11^ p/km (see for example DPF N2 #4, DPF N3 #3, #7, #9). In these cases the SCR system is downstream of the DPF and the emissions increase when the exhaust gas temperature has reached the appropriate range and urea is injected. Figure 5 shows two WHVCs one after the other (the first one with cold engine start). Although at the beginning there are almost no particles, when NO_x_ emissions decrease (due to urea injection), the SPN concentration increases. According to the literature, in addition to the formation of nitrates and sulfates [55], these particles originate from isocyanic acid polymerization, urea pyrolysis and urea micro-explosions [54]. This explains why in some cases the cold start SPN emissions are lower than the hot start. Note also the high concentration of sub-23 nm particles, indicating that the mean size of the formed solid particles is lower than 20 nm. 

### 4.3. Ambient Temperature 

**Figure 6:**
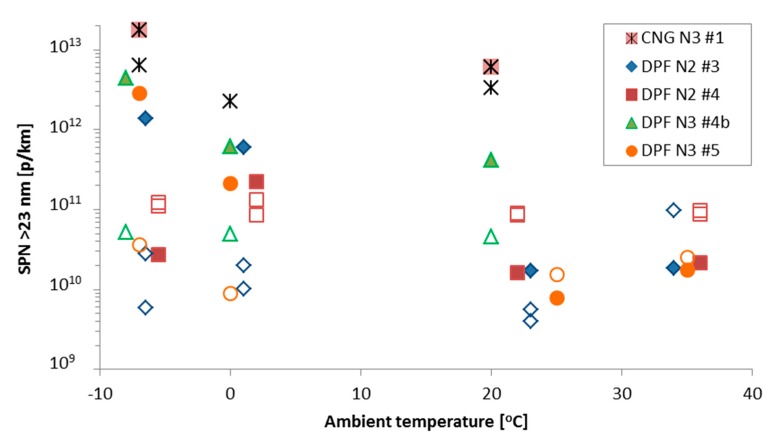
Urban phase with cold (solid symbols) or hot (open symbols) engine start at different ambient temperatures for different vehicles. Temperatures have been shifted ±1 °C for better visualization.

Figure 6 presents the effect of ambient temperature on SPN emissions. Beginning with the hot engine start (open symbols) of the DPF vehicles, the emission levels are relatively flat (i.e., similar levels within experimental uncertainty) indicating no influence of the ambient temperature. This is expected, as long as the cylinder, lubricant and after-treatment devices have reached their operation temperature, and as long as the engine strategy does not change (e.g., Exhaust Gas Recirculation (EGR)).

The urban SPN emissions with engine cold start have a tendency to increase with decreasing ambient temperature (solid symbols, Figure 6). According to the literature [35,75], the cold start particles emission can be due to: (i) higher engine out emissions (ii) semi-volatile material escaping oxidation, (iii) blow-out of loose non-volatile particle deposits, (iv) particles penetrating through small filter defects which close as the temperature rises. For light-duty vehicles it was suggested that the last assumption is the most probable: Small defects in the brick or in the mat employed to mount the brick in the canister result in reduced filtration efficiency. The lower canister temperature at −7 °C require prolonged thermal stabilization period, especially for the mounting material being in direct contact with the canister. 

However, the SPN emissions during cold start are not always higher at lower temperatures (see e.g., DPF N2 #4 of Figure 6 (or [52]). The DPF fill state plays the most important role and determines the efficiency of the DPF. For example, a study with two DPF equipped heavy-duty vehicles showed that the emissions immediately after a regeneration event were >5 × 10^12^ p/kWh and dropped to <1 × 10^11^ p/kWh after 5 test cycles [76], clearly demonstrating the importance of the soot cake of the DPF on its efficiency. The tests in this paper were not controlled in terms of DPF load state.

Regarding the CNG vehicle, there is an increase of emissions at 0 °C and −7 °C compared to 22 °C, but no difference between 0 °C and −7 °C. There are no studies discussing the effect of low temperature and cold start on SPN emissions of CNG vehicles. One possible explanation is the increased contribution of soot and lubricant due to the incomplete combustion. The smaller relative increase of the emissions at lower ambient temperatures is due to the higher increase of sub-23 nm particles. This indicates that the majority of the emissions at low ambient temperatures are due to lubricant particles (soot particles are usually >20 nm), based also on other studies that found that the non-volatile particles of CNG vehicles were composed mostly of ash from lubricating oil [77,78].

### 4.4. Urban, Rural, and Motorway Phases

Comparison of the emissions from the hot urban, rural and motorway phases shows that in most cases the hot urban emissions are higher than the rural and motorway phases (Figure 3). One would expect higher emissions at the motorway phase due to higher engine out emissions (as for cases CNG N3 #1, DPF N2 #1, DPF N3 #3). For example, for the CNG N3 #1, the combustion at the motorway phase might be rich and incomplete, resulting in higher emissions. For DPF vehicles the higher exhaust gas temperature passively regenerates the filter and consequently reduces the filter efficiency of the DPF. Moreover, at rural and motorway conditions, it is expected that urea is injected more time than during urban conditions; this could lead to higher SPN emissions as previously discussed. One probable explanation for the higher urban emissions is that the emissions are still relatively high due to the cold start (e.g., CNG N1 #1, CNG N3 #2, LNG N3 #1, DPF N2 #1, DPF N3 #1, DPF N3 #2). Another one is that the emissions are very low and the higher distances at the rural and motorway phases result in lower p/km (DPF N2 #4, DPF N3 #7). 

### 4.5. Correlation of Cycles

**Figure 7:**
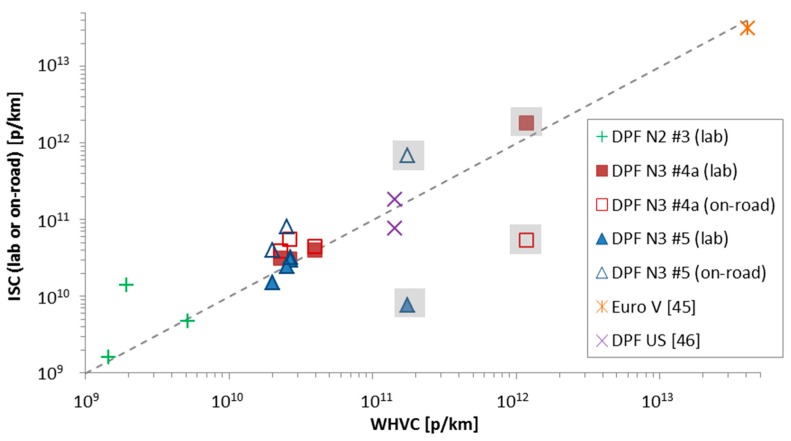
Correlation of different phases of WHVC and ISC cycles (cold start, urban, rural, motorway) in the laboratory and on the road. Symbols with grey background refer to urban cold start emissions.

For some vehicles it was possible to compare each phase (cold start, urban, rural, motorway) of the lab WHVCs to the lab and on-road ISC cycles. The correlation between different phases is presented in Figure 7. Additionally some tests from the literature were plotted [465,456]. One case was a Euro V truck [45] and the other was a 14.6 L truck retrofitted with a Continuously Regenerating Trap (CRT) that was tested with the Urban Dynamometer Driving Schedule (USSD) lab cycle and some on-road trips [46].

In general, there is an acceptable agreement between the different phases of different cycles (e.g., rural WHVC vs. rural ISC), but not always. For some cases the cold start emissions of the ISC cycles (points in grey background in Figure 7) are lower than the WHVC cold start emissions. This could be partly due to the smoother driving on the road. However, probably the DPF load state plays the most important role and determines the emission levels.

### 4.6. Regeneration

As soot accumulates in the DPF, there is a need for periodic regeneration (oxidation of the soot) in order to avoid clogging of the DPF or uncontrolled oxidation of the soot, which can potentially damage the DPF. In this case the increase of the temperature is initiated by the vehicle’s engine management system (e.g., post injection of fuel) and is called active regeneration. Under some operating conditions of the vehicle or engine and the assistance of NO_2_, the exhaust gas temperature is high enough to oxidize the soot (passive regeneration) [79]. Systems that do not need periodic regeneration are called continuously regenerating systems. Nevertheless, vehicles with continuously regenerating systems can have additionally a periodic regeneration trigger. Heavy-duty regulations require taking into account the emissions during regeneration for periodically regenerating systems in the certification value. 

**Figure 8:**
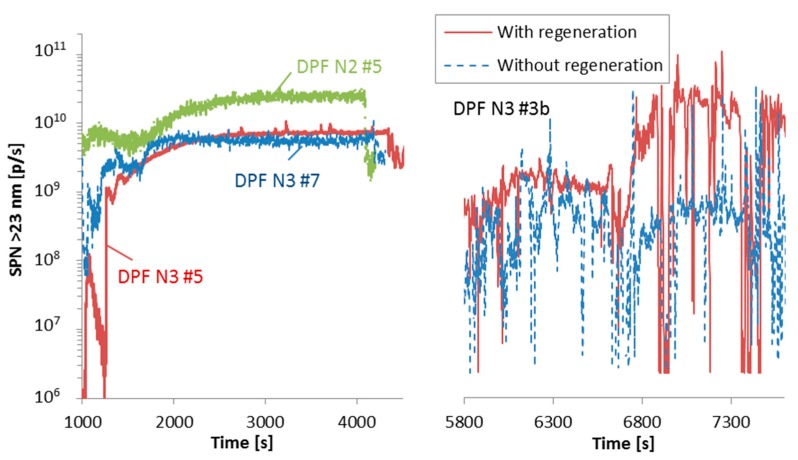
Examples of regeneration events. **Left**: Dashboard activated regeneration for three parked vehicles (time axis shifted for better visualization). Each color line refers to a different vehicle. **Right**: Two similar trips of vehicle DPF N3 #3b; one with regeneration, the other without.

To estimate the emissions during active regenerations under real conditions two cases were examined: Regeneration by dashboard activation with the vehicles parked and active regeneration during an on-road trip. Figure 8 presents these two regeneration cases. During the active regeneration of 3 parked vehicles (Figure 8 left panel) approximately 1.6 × 10^13^–5.1 × 10^13^ particles were emitted. The emissions remained high even after the regeneration as the DPF was empty. A similar study [57] that examined the active regeneration emissions of total particles (including volatiles) of two parked trucks found 4 × 10^16^ to 2 × 10^17^ particles. They mentioned though that the majority was nucleation mode particles, which were 2–4 orders of magnitude higher than the accumulation mode particles (which are close to the SPN emissions). 

During the on-road active regeneration (time 6700 s until 7400, Figure 8 right panel) approximately 1.1 × 10^13^ more particles were emitted compared to the non-regenerating trip. Note also that the emissions after the regeneration remained high. The motorway emissions of the two trips were 2.9 × 10^11^ p/km and 3.8 × 10^10^ p/km, respectively.

It is difficult to quantify the contribution of regeneration on SPN emissions. A very detailed study with Japanese 2009 heavy-duty trucks (similar to Euro VI) showed that the regenerating cycle contributed 43% (Truck with SCR) to 81% (truck without SCR) to the final weighted certification value [74]. These percentages increased to 88–99% when the emissions of the two cycles after regeneration were included. Thus, the rest of the tests (approximately 15) played no role and their exact value (typically <1 × 10^11^ p/km) was of minor importance. However, all these tests were conducted with hot engine start.

In this study, two of the vehicles (DPF N3 #4a and #5) were tested for more than one month in the laboratory and all SPN emissions were recorded (e.g., during warm-up, testing and active regeneration). The total SPN emissions divided by the total distance travelled for DPF N3#4a were 3.1 × 10^11^ p/km (or 2.6 × 10^11^ p/kWh) and for DPF N3 #5 were 6.6 × 10^11^ p/km (or 5.3 × 10^11^ p/kWh). Another vehicle that was tested for more than 1300 km, did not actively regenerate and the emissions were slightly lower than 1 × 10^11^ p/km. This means that the SPN emissions including the regeneration events remain at acceptable levels (i.e., close to the SPN Euro VI limit) and quite close to the values presented in Figure 2 (including the cold start).

### 4.7. Sub-23 nm Solid Particles

During the PMP investigation from 2008 to 2010 of the SPN methodology (>23 nm) for heavy-duty engines, there was some evidence that solid particles <23 nm were present; but not at levels that would justify any modification of the light-duty methodology [80]. Later review studies focused on light-duty vehicles and motorcycles [53,66,67], and only a few studies discussed about heavy-duty vehicles [81,82,83]. Some studies even showed that the measurements below 23 nm are prone to artifacts [53,67,82]. 

**Figure 9:**
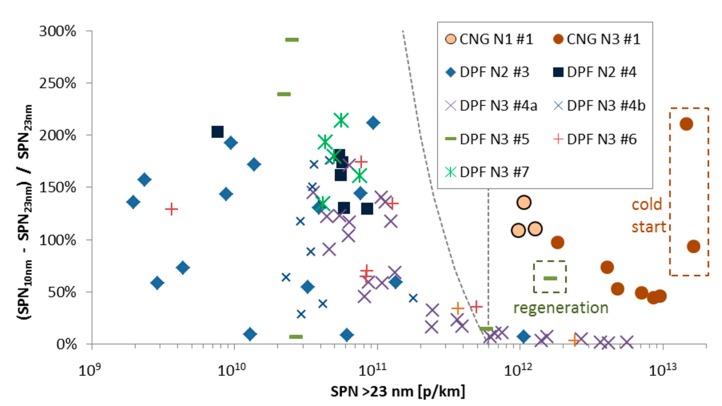
Sub-23 nm fraction (corrected for particle losses) in function of the SPN >23 nm emissions. Vertical dashed line indicates a limit of 6 × 10^11^ p/km for particles >23 nm in diameter (note that it is only an indication as the limit applies only to engines and is expressed in p/kWh). The other line indicates the same limit for particles >10 nm in diameter.

Figure 9 presents the sub-23 nm fraction of various heavy-duty vehicles in function of the SPN >23 nm emissions of the vehicles for various test cycles. Many of the measurements presented here were conducted in parallel with systems less prone to artifacts (catalytic strippers [53,67,82]), thus, the values presented should be reliable. The CNG vehicles have a high percentage (>50%) of sub-23 nm particles and one vehicle had even higher percentage at cold start (CNG N3 #1). The specific one though had <3000 km at the odometer and the contribution from the lubricant could be higher than the other vehicles [73].

Regarding the diesel vehicles equipped with DPF, for the same concentration range (>1 × 10^12^ p/km) the sub-23 nm fraction is very low (<10%). This is expected because DPFs have high filtration efficiency for all sizes [84]. However, at concentration levels <1 × 10^11^ p/km the sub-23 nm fraction reaches and sometimes exceeds 200%. These particles are probably urea decomposition particles, as discussed previously [54,55]. Solid soot cores below 20 nm have also been reported [83].

From Figure 9 is clear that the solid sub-23 nm fractions can be significant; however, the sub-23 nm absolute emission levels in most cases remain low. For example, DPF vehicles are still below the SPN limit even when including the sub-23 nm fraction (in Figure 9 all DPF points are below the curved dotted line). Thus, for regulatory purposes the current methodology still captures high emitters for most of the cases (i.e., a vehicle that passes with the 23 nm system would also pass with the 10 nm system and vice versa). A critical situation would be to have many vehicles in the area between the two dashed lines in Figure 9.

### 4.8. Total Particles

Although the purpose of the paper was to discuss solid particles, it should be mentioned that volatile (nucleation mode) particles can be orders of magnitude higher in concentration [47] and they are often measured in the exhaust plume of vehicles and in roadside environments [85]. Typical PN emission factors of heavy-duty diesel vehicles range from 1 × 10^14^ to 6 × 10^15^ p/km (see review [86]). Since that review, other studies have confirmed these levels [87], while for CNG or DPF equipped vehicles the levels were found similar [88] or lower [89,90,91] (around 2 × 10^11^ to 2 × 10^13^ p/km). 

The quantification of total PN concentration is difficult as their formation and concentration depends on many parameters, such as the after-treatment devices (e.g., [92]), the pre-conditioning and history of the vehicle (e.g., [93]), the fuel and the lubricant used (e.g., [94]), the ambient conditions (e.g., [95]), and the amount of soot present, since this promotes the competing process of condensation and adsorption instead of nucleation [96]. The regulated method uses constant volume sampling which means low dilution (around 6:1) at high exhaust flow rates and high dilution at low exhaust flow rates (>30:1). In the atmosphere the dilution process is the opposite: for example, in one chasing study 14 m from a light-duty vehicle, the dilution varied from 1800:1 at 50 km/h to 7000:1 at 120 km/h [97]. In addition to the concerns regarding the representativeness of the regulated methodology to quantify total PN emissions, the different designs and operational parameters among the test facilities increase the variability of their results. The reason is that, even when using the conditions allowed by the regulation, concentrations that differ orders of magnitude can be obtained [98]. Finally, the transfer line from the vehicle to the dilution tunnel can be a source of artifacts due to release of stored materials from previous tests and/or vehicles [99].

Dedicated designs of dilution systems to measure directly at the tailpipe [100] can estimate the formation “potential” of the nucleation mode, i.e., they can reproduce the trends, including those caused by differences in vehicle speed and engine load, engine and after-treatment technology, as well as fuel and lubricant composition [100,101]. However, a review study showed that the number concentration of the nucleation mode in the laboratory was generally lower by a factor of 2–10 from the atmosphere [101]. One explanation for the lower concentration in the laboratory was the choice of dilution parameters (dilution ratio 12, dilution temperature 32 °C, relative humidity <5%). The agreement was better when the laboratory sampling conditions matched those encountered on-road [95]. When the sampling parameters (e.g., dilution ratio or dilution temperature) are not appropriate, the measured particle number emissions might not be representative of the actual emissions on the road [102].

**Figure 10:**
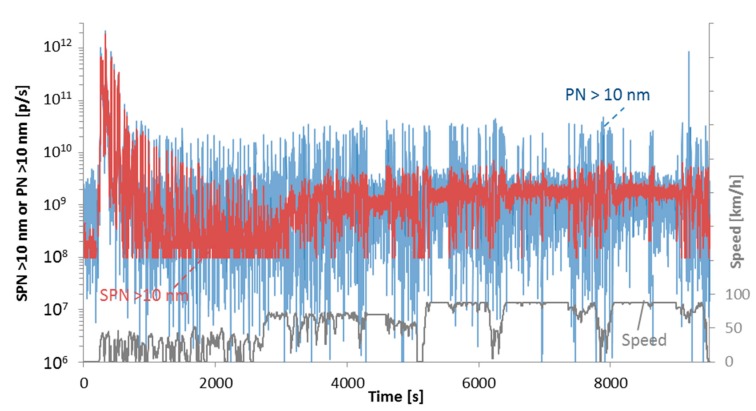
SPN and PN (>10 nm) emissions from the dilution tunnel (CVS) during an ISC cycle at −7 °C. Vehicle DPF N3 #4b.

Figure 10 shows an example of solid and total particles, both above 10 nm with vehicle DPF N3 #4a. The total PN concentration was measured with an Engine Exhaust Particle Sizer (EEPS, TSI model 3090, Shoreview, MN, USA). During the cold start the instruments agree within 15%, however at the rest urban part the total particles are >2 times higher in concentration, but as the solid particle concentration increases the difference of the two systems decreases to 40% (motorway phase). At the specific example the cold start emissions were around 6 × 10^12^ p/km, while at the rest of the cycle the emissions were around 8 × 10^10^ p/km. This test, which was done at −7 °C, didn’t increase the exhaust gas temperature at high levels to induce a strong nucleation mode.

**Figure 11:**
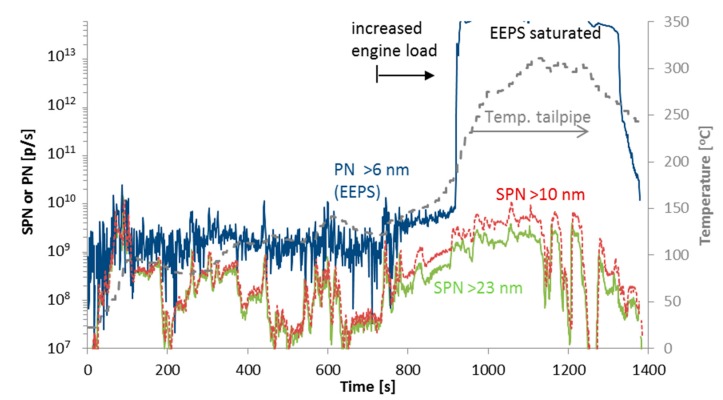
SPN and total PN emissions during a test with different engine loads and speeds to induce nucleation mode particles. Vehicle DPF N3 #4b.

Figure 11 shows SPN and total PN emissions during steady state points with different engine loads and consequently exhaust gas temperatures. At the beginning of the test, the difference between total and solid PN emissions is relatively small (see first part of Figure 11). However, when the exhaust gas temperature increases, a high concentration of nucleation mode particles is measured (see last part of Figure 11). This is attributed to the formation of sulfuric acid particles due to the high conversion of SO_2_ to SO_3_ at the catalyst of the vehicle (see [82,92,93,97,103]). Note that the SPN concentrations remained at relatively low levels. The SPN emissions were <10^11^ p/km, while total PN exceeded 10^15^ p/km.

### 4.9. Strengths and Limitations

This study is one of the first to investigate and summarize solid particle emissions from Euro VI heavy-duty vehicles, quantify their sub-23 nm fraction, discuss the effect and contribution of cold start, low ambient temperature, and regeneration events. The vehicles tested were Euro VI compliant and thus should be representative of near future emissions. Existing emission factors could be updated based on the findings of this study, especially for natural gas vehicles and cold start contribution. As there is no in-service conformity (ISC) requirement for particle emissions yet, improvements might be seen in the future, especially for gas engines, when they will have to comply with on-road limits. Another drawback of the study is that the results are based on European vehicles only and in some cases only one repetition was available. Although the fact that the tests were conducted in many locations by different companies enhances the validity and robustness of the findings; however, it does not allow a strict comparison between the different vehicles, as in many cases the protocols were different. Finally, this study only touched total particle emissions which are important for air quality monitoring purposes. Nevertheless, this study is a step in production of solid particle emission factors of modern Euro VI vehicles and might help emission inventories and air quality modeling for the estimation of contribution of road traffic to air pollution.

## 5. Conclusions

European regulation requires the measurement of Solid Particle Number (SPN) emissions with diameter >23 nm for heavy-duty engines. Additionally, the heavy-duty vehicles have to be checked for in-service conformity (ISC) with Portable Emission Measurement Systems (PEMS). This study presented the SPN emissions of 24 diesel, CNG or LNG vehicles (8 of them from the literature) on the road and in the laboratory driving realistic ISC cycles.

The SPN emissions ranged from 8 × 10^9^ to 7 × 10^11^ p/km for the diesel DPF equipped vehicles and 3.3 × 10^11^ to 4.5 × 10^12^ p/km for the CNG and LNG vehicles. The majority of the SPN were emitted during the cold start for most of the diesel vehicles. The ambient temperature had a significant effect only during cold start, but the effect was dependent on the DPF fill state.

Active regeneration events (vehicles parked or during driving) resulted in increased emissions. The contribution in SPN was 1.1 × 10^13^–5.1 × 10^13^ particles. Based on these vehicles, the weighted emissions including regeneration events were close or below the SPN limit.

The sub-23 nm fraction was significant: >50% for CNG engines (emission levels >1 × 10^12^ p/km) and up to 200% for diesel engines for emission levels <1 × 10^11^ p/km. Although the regulated SPN methodology can still distinguish low or high emitters regardless of the sub-23 nm fraction, monitoring this fraction is recommended in order to avoid situations that the methodology is not efficient.

## Figures and Tables

**Table 1:** Details of test fleet.

Nomenclature	Fuel	Category	Description	Engine Size ^1^ (L)	Power ^1^ (kW)
CNG N1 #1	CNG	N1	Rigid truck	3	100
CNG N3 #1	CNG	N3	Refuse collection	9	185
CNG N3 #2	CNG	N3	Rigid truck	8	220
CNG N3 #3	CNG	N3	Refuse collection	9	250
CNG M3 #1	CNG	M3	City bus	13	230
LNG N3 #1	LNG	N3	Tractor semi trailer	9	300
LNG N3 #2	LNG	N3	Tractor semi trailer	9	250
DPF N2 #1	Diesel	N2	Rigid truck	8	200
DPF N2 #2	Diesel	N2	Rigid truck	5	180
DPF N2 #3	Diesel	N2	Rigid truck	5	180
DPF N2 #4	Diesel	N2	Rigid truck	5	155
DPF N2 #5	Diesel	N2	Rigid truck	5	180
DPF N2 #6	Diesel	N2	Rigid truck	8	180
DPF N3 #1	Diesel	N3	Tractor semi trailer	13	340
DPF N3 #2	Diesel	N3	Tractor semi trailer	13	340
DPF N3 #3	Diesel	N3	Tractor semi trailer	13	320
DPF N3 #4	Diesel	N3	Tractor semi trailer	13	340
DPF N3 #5	Diesel	N3	Tractor semi trailer	13	340
DPF N3 #6	Diesel	N3	Tractor semi trailer	13	340
DPF N3 #7	Diesel	N3	Rigid truck	8	240
DPF N3 #8	Diesel	N3	Rigid truck	11	270
DPF N3 #9	Diesel	N3	Rigid truck	8	180
DPF N3 #10	Diesel	N3	Crane	11	240
DPF M3 #1	Diesel	M3	Bus	11	330

^1^ Values are approximate in order not to disclose the vehicle model. CNG: Compressed Natural Gas; LNG: Liquefied Natural Gas; DPF: Diesel Particulate Filter.

**Table 2:** Test details. More information in the main text.

Nomenclature	Tested by	Cycle	Place	T_amb_ (°C)	Test Weight ^1^ (t)	Add. Testing
CNG N1 #1	ACEA	ISC	On-road	24	2.5	<23 nm
CNG N3 #1	JRC	ISC	Lab	22	20	<23 nm, T
CNG N3 #2	ACEA	ISC	On-road	29	27	
CNG N3 #3	STA [59]	WHVC	Lab	23	20	
CNG M3 #1	ACEA	ISC	On-road	21	20	
LNG N3 #1	TNO [58]	ISC	On-road	0	31	
LNG N3 #2	TNO [58]	ISC	On-road	3	31	
DPF N2 #1	ACEA	ISC	On-road	25	12	
DPF N2 #2	ACEA	ISC	On-road	24	9	
DPF N2 #3	JRC	ISC	Lab	23	9	<23 nm, T, C
DPF N2 #4	JRC	ISC	Lab	22	9	<23 nm, T
DPF N2 #5	JRC	ISC	Lab	20	12	Reg.
DPF N2 #6	STA [59]	WHVC	Lab	23	10	
DPF N3 #1	ACEA	ISC	On-road	20	26	
DPF N3 #2	ACEA	ISC	On-road	28	22	
DPF N3 #3a,b	JRC [45,52]	ISC	On-road	15	27	Reg.
DPF N3 #4a,b	JRC	ISC	Lab	20	27	<23 nm, T, C
DPF N3 #5	JRC	ISC	Lab	20	27	<23 nm, T, C, Reg.
DPF N3 #6	JRC	WHVC	Lab	21	27	<23 nm
DPF N3 #7	JRC	WHVC	Lab	23	16	<23 nm
DPF N3 #8	STA [59]	WHVC	Lab	23	18	
DPF N3 #9	STA [59]	WHVC	Lab	23	15	
DPF N3 #10	STA [59]	WHVC	Lab	23	21	
DPF M3 #1	ACEA	ISC	Lab	24	18	

^1^ Payload approximately 50–60% in all cases. Additional testing: T = Low ambient temperature tests, C = Cycles comparisons, Reg. = Regeneration, <23 nm = Sub-23 nm investigation. ACEA: European Automobile Manufacturers’ Association; ISC: in-service conformity; JRC: European Commission’s Joint Research Centre; STA: Swedish Transport Agency; TNO: The Netherlands Organization for Applied Scientific Research; WHVC: Worldwide Harmonized Vehicle Cycle.

## Abbreviations

ACEA	European Automobile Manufacturers Association
APC	AVL Particle Counter
CNG	Compressed Natural Gas
COPERT	Computer Programme to calculate emissions from road transport
CPC	Condensation Particle Counter
CRT	Continuously Regenerating Trap
CVS	Constant Volume Sampling
DOC	Diesel Oxidation Catalyst
DPF	Diesel Particulate Filter
EEPS	Engine Exhaust Particle Sizer
EGR	Exhaust Gas Recirculation
EU	European Union
GDI	Gasoline Direct Injection
ISC	In-Service Conformity
HBEFA	Handbook Emission Factors for Road Transport
JRC	Joint Research Centre
LNG	Liquefied Natural Gas
MOVES	Motor Vehicle Emission Simulator
NPET	Nanoparticle Emission Tester
NTE	Not-To-Exceed
PCRF	Particle Concentration Reduction Factor
PEMS	Portable Emissions Measurement System
PFI	Port Fuel Injection
PM	Particulate Matter
PMP	Particle Measurement Programme
PN	Particle Number
RDE	Real Driving Emissions
SCR	Selective Catalytic Reduction
SPN	Solid Particle Number
STA	Swedish Transport Agency
TNO	Netherlands Organization for Applied Scientific Research
TWC	Three-Way Catalyst
USA	United States of America
USSD	Urban Dynamometer Driving Schedule
VELA	Vehicle Emissions Laboratory
VPR	Volatile Particle Remover
WHTC	Worldwide Harmonized Transient Cycle
WHVC	Worldwide Harmonized Test Cycle
